# Pregnancy-Induced High Plasma Levels of Soluble Endoglin in Mice Lead to Preeclampsia Symptoms and Placental Abnormalities

**DOI:** 10.3390/ijms22010165

**Published:** 2020-12-26

**Authors:** Lucía Pérez-Roque, Elena Núñez-Gómez, Alicia Rodríguez-Barbero, Carmelo Bernabéu, José M. López-Novoa, Miguel Pericacho

**Affiliations:** 1 Institute of Biomedical Research of Salamanca (IBSAL), 37007 Salamanca, Spain; luciap@usal.es (L.P.-R.); elena_biom2@usal.es (E.N.-G.); barberoa@usal.es (A.R.-B.); jmlnovoa@usal.es (J.M.L.-N.); 2 Renal and Cardiovascular Physiopathology Unit, Department of Physiology and Pharmacology, University of Salamanca, 37007 Salamanca, Spain; 3 Centro de Investigaciones Biológicas Margarita Salas, Consejo Superior de Investigaciones Científicas (CSIC) and Centro de Investigación Biomédica en Red de Enfermedades Raras (CIBERER), 28040 Madrid, Spain; bernabeu.c@cib.csic.es

**Keywords:** soluble endoglin, placenta, preeclampsia, murine model

## Abstract

Preeclampsia is a pregnancy-specific disease of high prevalence characterized by the onset of hypertension, among other maternal or fetal signs. Its etiopathogenesis remains elusive, but it is widely accepted that abnormal placentation results in the release of soluble factors that cause the clinical manifestations of the disease. An increased level of soluble endoglin (sEng) in plasma has been proposed to be an early diagnostic and prognostic biomarker of this disease. A pathogenic function of sEng involving hypertension has also been reported in several animal models with high levels of plasma sEng not directly dependent on pregnancy. The aim of this work was to study the functional effect of high plasma levels of sEng in the pathophysiology of preeclampsia in a model of pregnant mice, in which the levels of sEng in the maternal blood during pregnancy replicate the conditions of human preeclampsia. Our results show that wild type pregnant mice carrying human sEng-expressing transgenic fetuses (f*WT*(*hsEng^+^*)) present high plasma levels of sEng with a timing profile similar to that of human preeclampsia. High plasma levels of human sEng (hsEng) are associated with hypertension, proteinuria, fetal growth restriction, and the release of soluble factors to maternal plasma. In addition, f*WT*(*hsEng^+^*) mice also present placental alterations comparable to those caused by the poor remodeling of the spiral arteries characteristic of preeclampsia. In vitro and ex vivo experiments, performed in a human trophoblast cell line and human placental explants, show that sEng interferes with trophoblast invasion and the associated pseudovasculogenesis, a process by which cytotrophoblasts switch from an epithelial to an endothelial phenotype, both events being related to remodeling of the spiral arteries. Our findings provide a novel and useful animal model for future research in preeclampsia and reveal a much more relevant role of sEng in preeclampsia than initially proposed.

## 1. Introduction

Preeclampsia is a pregnancy-specific disease which affects about 3–5% of all pregnancies worldwide [1]. It is the major cause of maternal, fetal, and neonatal mortality in developed nations. Preeclampsia is characterized by hypertension (>140/90 mmHg) and proteinuria (>300 mg/24 h), appearing mainly after 20 weeks of pregnancy. In addition, preeclamptic women can present other alterations such as endothelial dysfunction, as well as disorders of the liver, kidney, brain, and clotting system [1,2]. Therefore, preeclampsia is considered to be a complex and multisystem disease [3].

Despite the abundant literature about the pathophysiology of preeclampsia, its molecular pathogenesis is mostly unknown. It is widely accepted that the placenta is key to the pathogenesis of this condition, as evidenced by the rapid disappearance of symptoms after delivery, and their persistence while the placenta is not removed [4,5]. Strong experimental evidence suggests that placental ischemia, resulting from the inappropriate remodeling of the maternal spiral arteries, stimulates the release of soluble factors causing hypertension, renal damage, and other preeclampsia-associated alterations [6,7]. Among the various factors released from the preeclamptic placenta, there has been increased interest in soluble endoglin (sEng). 

Human sEng can be generated upon a metalloprotease-mediated proteolytic cleavage acting on the extracellular domain of the full-length membrane bound endoglin (Eng) [8]. Full-length endoglin, a 180 kDa disulfide-linked homodimer, is a type I integral membrane glycoprotein with a large extracellular domain (561 amino acids), a single hydrophobic transmembrane domain, and a short cytosolic tail [9]. Eng is very abundant in endothelial cells and is also present in other cell types, including trophoblasts and syncytiotrophoblasts [10,11,12]. Shedding of sEng from these cell types in the placenta has been described to be mediated by the matrix metalloprotease (MMP)-14 [13,14,15,16]. Of note, expression levels and enzymatic activity of MMP-14 are increased in various conditions related to endothelial injury, activation, inflammation and senescence, as well as in pregnancy [17,18,19,20,21]. Several lines of evidence support a functional role of sEng in cardiovascular conditions and diseases, including endothelial dysfunction, anti-angiogenic activity, hypertension, increased vascular permeability, vascular remodeling, and inflammation-associated leukocyte adhesion and extravasation [8,14,20,22,23,24,25,26,27]

Several observations have linked high levels of sEng and other vasoactive factors in maternal circulation with clinical manifestations of preeclampsia [18,28,29,30]. Thus, sEng levels in plasma, as well as the ratio between plasma levels of soluble fms-like kinase 1 (sFlt1), a soluble form of the vascular endothelial growth factor (VEGF) receptor type-1, and placental growth factor (PlGF, a member of the VEGF family) are increased weeks before the onset of preeclampsia [17,31,32]. Furthermore, an increase of these values correlates with a higher severity and a worse prognosis of preeclampsia [17,18,33,34,35]. For this reason, sEng and sFlt1/PlGF ratio have been proposed to be useful biomarkers for diagnosis and prognosis of preeclampsia [17,36].

Although the correlation between increased levels of sEng and preeclampsia has been repeatedly described in the literature, there are only a few reports that have analyzed the putative role of sEng in the symptoms characteristic of preeclampsia or in the onset of this disease [14,18,37,38]. In this regard, we previously reported that mice continuously exposed to high circulating levels of sEng showed preeclampsia-like symptoms such as hypertension and proteinuria in the absence of pregnancy [14]. In addition, we postulated that the sEng-induced effect in hypertension was mediated by an upregulation of bone morphogenetic protein 4 (BMP4) [26]. Unfortunately, none of these studies phenocopied the maternal syndrome of preeclampsia, since they were not performed in mice with pregnancy-induced high circulating levels of sEng. Thus, our aim, in this work, was to study the role of high plasma levels of sEng in the pathophysiology of preeclampsia in a model of pregnant mice, in which the levels of sEng in the maternal blood during pregnancy replicate the conditions of human preeclampsia. Our results show that wild type pregnant mice carrying human sEng-expressing transgenic fetuses present high plasma levels of human sEng (hsEng) and clinical manifestations associated with the preeclampsia condition. This animal model also shows placental alterations such as oxidative stress, inflammation, and reduced pseudovasculogenesis, a process by which cytotrophoblasts switch from an epithelial to an endothelial phenotype, crucial for remodeling of the spiral arteries and supply of maternal blood. These findings provide a better understanding of the role of sEng in the pathophysiology of preeclampsia and have potential implications in preventive and therapeutic approaches for this condition. 

## 2. Results

### 2.1. Wild Type Pregnant Females with hsEng^+^ Fetuses Show High Plasma Levels of Soluble Eng

**Figure 1:**
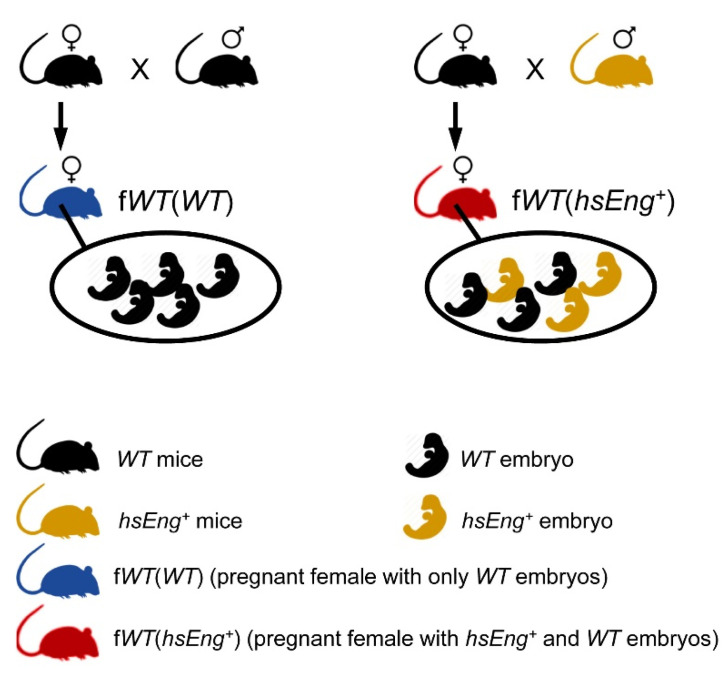
Description of the animal model. Wild type (WT) female mice crossed with transgenic male mice overexpressing human soluble endoglin (*hsEng^+^*), named as f*WT*(*hsEng^+^*), carry both *WT* and *hsEng^+^* embryos. *WT* female mice crossed with *WT* male mice, named as f*WT*(*WT*), carry only *WT* embryos.

**Figure 2:**
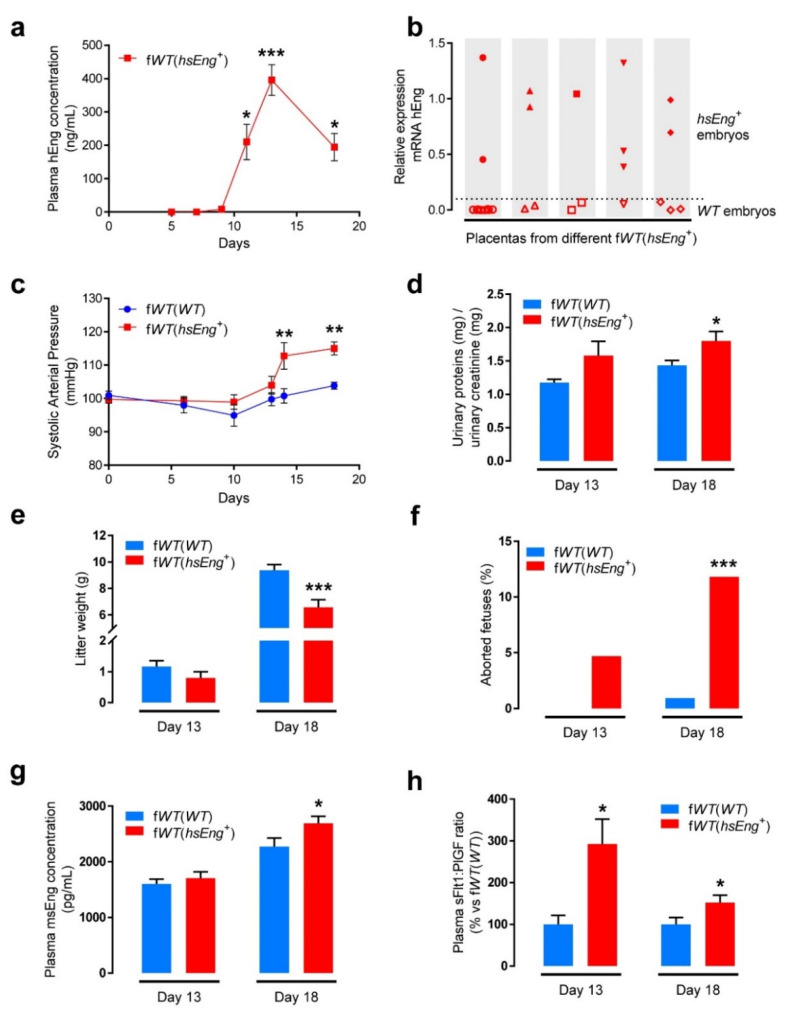
Pregnant f*WT*(*hsEng^+^*) mice show high plasma levels of soluble endoglin (sEng) and signs of preeclampsia. (**a**) Plasma levels of hsEng in f*WT*(*hsEng^+^*) mice (*n* = 6) at different time points during gestation; (**b**) qRT-PCR analysis of hEng mRNA expression in placentas from 5 different f*WT*(*hsEng^+^*) mice litters. Each column represents one litter, and each value corresponds to one placenta from that litter. Placentas with higher levels of hEng expressions are inferred to be from *hsEng^+^* embryos, whereas placentas with almost no expression are inferred to be from WT embryos; (**c**) Systolic arterial pressure of f*WT*(*WT*) (*n* = 9) and f*WT*(*hsEng^+^*) (*n* = 12) mice at different time points during gestation; (**d**) Urinary protein excretion, normalized to urinary creatinine concentration, of f*WT*(*WT*) and f*WT*(*hsEng*^+^) at Day 13 (f*WT*(*WT*) *n* = 8 and f*WT*(*hsEng^+^*) *n* = 9) and Day 18 (f*WT*(*WT*) *n* = 19 and f*WT*(*hsEng^+^*) *n* = 21) of gestation; (**e**) Weight of the litters from f*WT*(*WT*) and f*WT*(*hsEng^+^*) mice at Day 13 (f*WT*(*WT*) *n* = 5 litters and f*WT*(*hsEng^+^*) *n* = 6 litters), and Day 18 (f*WT*(*WT*) *n* = 18 litters and f*WT*(*hsEng^+^*) *n* = 22 litters) of gestation; (**f**) Percentage of aborted fetuses over total fetuses observed on Day 13 or Day 18 of gestation; (**g**) Plasma levels of murine sEng in f*WT*(*WT*) and f*WT*(*hsEng^+^*) at Day 13 (f*WT*(*WT*) *n* = 12 and f*WT*(*hsEng^+^*) *n* = 15) and Day 18 (f*WT*(*WT*) *n* = 10 and f*WT*(*hsEng*^+^) *n* = 13) of gestation; (**h**) sFlt1:PlGF ratio in plasma from f*WT*(*WT*) and f*WT*(*hsEng^+^*) mice at Day 13 (f*WT*(*WT*) *n* = 6 and f*WT*(*hsEng^+^*) *n* = 7) and Day 18 (f*WT*(*WT*) *n*=12 and f*WT*(*hsEng^+^*) *n* = 11) of gestation. Mean ± SEM are displayed. * *p* < 0.05, ** *p* < 0.01, and *** *p* < 0.001 of f*WT*(*hsEng^+^*) vs. f*WT*(*WT*).

The aim of this study was to develop an animal model with increased levels of human sEng during pregnancy, mimicking the profile of sEng plasma levels in preeclampsia. For this purpose, a previously reported transgenic mouse line which overexpresses human sEng (*hsEng^+^*) was used [14]. *Wild type* (*WT*) female mice were crossed with hsEng*^+^* male mice, so the WT pregnant females bore both *WT* and *hsEng^+^* fetuses, and hereinafter are referred to as f*WT*(*hsEng^+^*). In parallel, *WT* female mice were crossed with *WT* male mice, resulting in pregnant *WT* females bearing only *WT* fetuses and named f*WT*(*WT*) (Figure 1). Analysis of plasma samples from pregnant f*WT*(*hsEng^+^*) mice showed increased levels of hsEng from the 11th day of gestation and this increase was sustained until the end of pregnancy, similar to human preeclampsia (Figure 2a). As expected, no hsEng was detected in pregnant f*WT*(*WT*) mice. 

The overall association between plasma levels of hsEng in f*WT*(*hsEng^+^*) females and the growth of fetuses, strongly suggested a fetal source for hsEng, probably from the fetal placenta. Thus, we checked the expression of hsEng in placentas of five different f*WT*(*hsEng^+^*) mice litters. We observed that expression levels of hsEng were variable, with some placentas showing high levels (likely, placentas from *hsEng^+^* embryos) and others with low levels and almost no expression, likely from WT embryos (Figure 2b). 

### 2.2. Pregnant fWT(hsEng^+^) Mice Present Symptoms and Biomarkers of Preeclampsia

Next, we evaluated the relationship between high plasma levels of hsEng and one of the hallmarks of preeclampsia, i.e., increased arterial pressure. We observed that the arterial pressure was higher in f*WT*(*hsEng^+^*) than in f*WT*(*WT*) pregnant mice from the 13th day of gestation until the end of pregnancy (Figure 2c). From there on, we took measurements at two specific time points as follows: (i) on the 13th day, when the highest levels of sEng were detected and significant differences in blood pressure started to be observed and (ii) on the 18th day, immediately before the end of the gestation (21 days), to ensure records even in case of premature birth, and because in f*WT*(*hsEng^+^*) mice many fetuses were already necrotic at this time point.

Another characteristic clinical manifestation of preeclampsia is proteinuria. We found that the urinary protein excretion was significantly higher in f*WT*(*hsEng^+^*) than f*WT*(*WT*) at the 18th day of gestation, whereas this difference was not significant at the 13th day of gestation (Figure 2d). We also assessed whether proteinuria was associated with structural or functional renal alterations. Glomerular filtration rate, measured as creatinine clearance, was similar in f*WT*(*hsEng*^+^) as compared with f*WT*(*WT*) mice at the end of gestation (Figure S1a). In addition, upon hematoxylin and eosin (H&E) staining of renal tissue at the end of gestation, no relevant alterations in kidney structure were observed in either f*WT*(*hsEng^+^*) or f*WT*(*WT*) females (Figure S1b).

Because fetal growth is restricted in preeclampsia, we analyzed this parameter in our model. Of note, a direct comparison about fetal growth restriction between human and mouse pregnancy is not easy. Humans are usually uniparous and fetal growth restriction can only result in a smaller fetus. However, mice, being multiparous, can present the following different outcomes: (i) proportional reduction of the size in all the offspring; (ii) reduction of size in part of the offspring, while other fetuses remain normal; and (iii) demise of some fetuses, maintaining a normal size in the others. For this reason, we considered that, in mice, the analysis of total litter weight was the most appropriate parameter to assess fetal growth restriction. Litter weight was significantly lower at Day 18 of pregnancy in f*WT*(*hsEng^+^*) than in f*WT*(*WT*) females (with a reduction of 33%), but this difference was not significant at Day 13 (Figure 2e). The observed effect on litter weight was due to both a reduction in the size of viable fetuses and a reduction in the number of viable fetuses (Figure S2). Thus, the number of aborted fetuses at Day 18 was markedly higher (10-fold change) in f*WT*(*hsEng^+^*) than in f*WT*(*WT*) pregnant females, since this effect was evident even at Day 13 (Figure 2f).

Next, we assessed the levels of some biomarkers that are characteristic of preeclampsia. Plasma levels of endogenous murine sEng (msEng), were significantly higher in f*WT*(*hsEng^+^*) than in f*WT*(*WT*) pregnant mice at the end of gestation (Day 18), whereas no significant differences were observed at Day 13 (Figure 2g). Because the ratio sFlt1/PlGF in plasma has been reported to be an accurate predictor of preeclampsia [39], we also analyzed plasma levels of sFlt1 and PlGF in our animal model. We observed a significantly higher sFlt1/PlGF ratio in f*WT*(*hsEng*^+^) than f*WT*(*WT*) pregnant mice after 13 and 18 days of gestation (Figure 2h). These results suggest that pregnancy-induced high levels of plasma sEng contribute to the release of vasoactive soluble factors in our animal model.

Taken together, these data suggest that *WT* pregnant female mice carrying hsEng*^+^* fetuses, f*WT*(*hsEng^+^*), are a good model to study the role of sEng in preeclampsia because they present the most important symptoms and specific biomarkers of the disease, as well as a temporal development during pregnancy that resembles what happens in human preeclampsia. Additionally, these evidences demonstrate that increased plasma sEng triggers some of the symptoms of preeclampsia, suggesting sEng as an active factor in the progression of the disease.

### 2.3. Presence of Soluble Endoglin Is Associated with Placental Alterations

**Figure 3:**
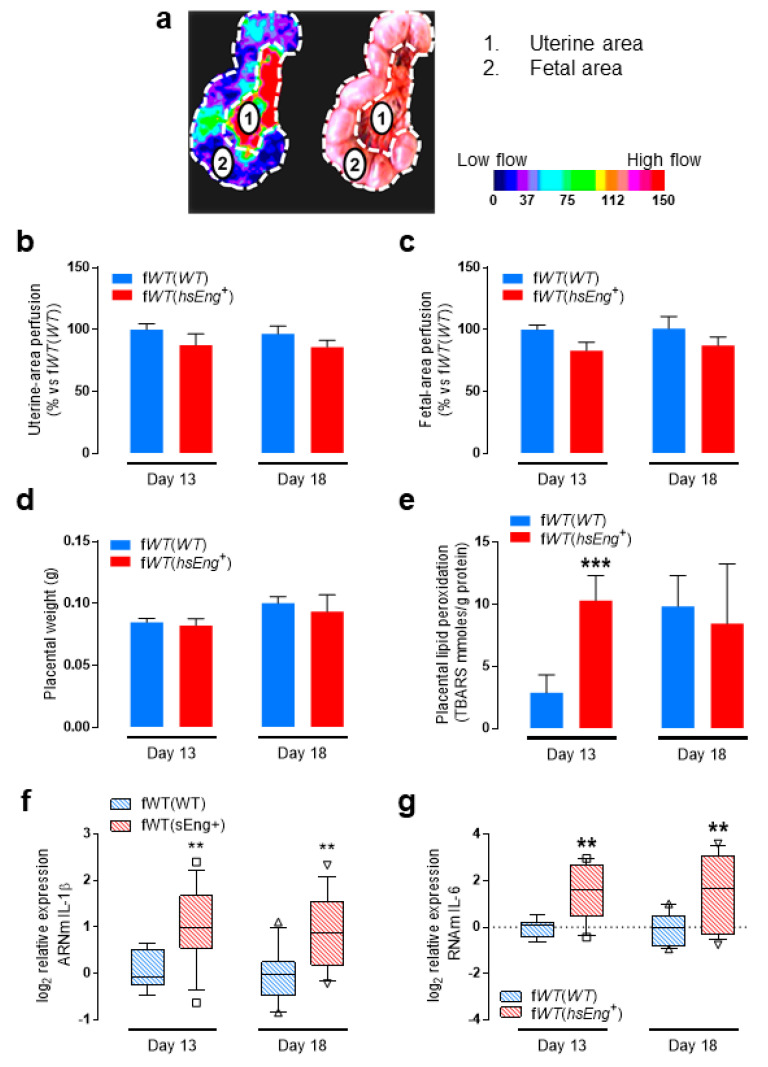
Pregnant f*WT*(*hsEng^+^*) mice present placental alterations. (**a**) Representative image of the analysis of placental and fetal perfusion by laser Doppler imaging. For each scan, the algorithm builds up a color-coded image representing tissue perfusion (left) and records a color photograph at the same time (right). Perfusion was determined in two different areas defined by color photograph, i.e., uterine area and fetal area. As shown in the scale, the pseudocolor ranges from left to right, therefore, blue represents the lowest perfusion and red the highest perfusion; (**b**) Quantification of the uterine-area perfusion from f*WT*(*WT*) and f*WT*(*hsEng^+^*) mice at Day 13 (f*WT*(*WT*) *n* = 7 and f*WT*(*hsEng^+^*) *n* = 7) and Day 18 ((f*WT*(*WT*) *n* = 11 and f*WT*(*hsEng^+^*) *n* = 15) of gestation; (**c**) Quantification of the fetal-area perfusion from f*WT*(*WT*) and f*WT*(*hsEng^+^*) mice at Day 13 (f*WT*(*WT*) *n* = 7 and f*WT*(*hsEng^+^*) *n* = 7) and Day 18 (f*WT*(*WT*) *n* = 11 and f*WT*(*hsEng^+^*) *n* = 15) of gestation; (**d**) Weight of the placentas from f*WT*(*WT*) and f*WT*(*hsEng^+^*) mice at Day 13 (f*WT*(*WT*) *n* = 7 and f*WT*(*hsEng^+^*) *n* = 7) and Day 18 (f*WT*(*WT*) *n* = 9 and f*WT*(*hsEng^+^*) *n* = 8] of gestation; (**e**) Lipid peroxidation, measured as presence of thiobarbituric acid-reactive substances (TBARS), of placentas from f*WT*(*WT*) and f*WT*(*hsEng^+^*) mice at Day 13 (f*WT*(*WT*) *n* = 20 and f*WT*(*hsEng^+^*) *n* = 21) and Day 18 (f*WT*(*WT*) *n* = 13 and f*WT*(*hsEng^+^*) *n* = 12) of gestation; (**f**) qRT-PCR analysis of IL-1β expression in the placentas from f*WT*(*WT*) and f*WT*(*hsEng^+^*) mice at Day 13 (f*WT*(*WT*) *n* = 12 and f*WT*(*hsEng^+^*) *n* = 13) and Day 18 (f*WT*(*WT*) *n* = 12 and f*WT*(*hsEng^+^*) *n* = 15) of gestation; (**g**) qRT-PCR analysis of IL-6 expression in the placentas from f*WT*(*WT*) and f*WT*(*hsEng^+^*) mice at Day 13 (f*WT*(*WT*) *n* = 12 and f*WT*(*hsEng^+^*) *n* = 13) and Day 18 (f*WT*(*WT*) *n* = 12 and f*WT*(*hsEng^+^*) *n* = 15) of gestation. Mean ± SEM are displayed. ** *p* < 0.01 and *** *p* < 0.001 of f*WT*(*hsEng^+^*) vs. f*WT*(*WT*).

Because pathophysiology of preeclampsia is associated with placental alterations, we analyzed whether sEng could induce these effects in our animal model. In preeclampsia, alterations in remodeling of uterine arteries reduce blood flow to the placenta and induce placental hypoxia [35,36]. A visual display of the mouse uterus shows the necklace-bead arrangement of the mouse fetuses with their placentas and a large area including the uterine arteries. Thus, we measured fetal and uterine blood flow, as shown in Figure 3a, by laser Doppler flowmetry in pregnant mice. No significant differences, in both uterine and fetal blood flow, were found between f*WT*(*hsEng^+^*) and f*WT*(*WT*) mice at either 13 or 18 days of pregnancy (Figure 3b,c). In addition, placental weight was similar in f*WT*(*hsEng^+^*) and f*WT*(*WT*) pregnant mice at Days 13 and 18 (Figure 3d). Together, these results do not support a causal relationship between the high levels of plasma sEng and placental growth restriction.

Oxidative stress and inflammation also play an important role in the pathophysiology of preeclampsia [37,38,39]. We found that placentas of f*WT*(*hsEng^+^*) mice showed a significantly higher (four-fold change) lipid peroxidation than those of f*WT*(*WT*) animals at Day 13; but not at Day 18 of pregnancy (Figure 3e). The lack of difference at Day 18 was due to the expected late increase of this parameter in f*WT*(*WT*) animals, suggesting an abnormal early increase of placental lipid peroxidation in f*WT*(*hsEng^+^*) as compared with f*WT*(*WT*) animals (Figure 3e). Moreover, we observed that the expression of inflammatory cytokine genes, such as IL-1β and IL-6, was higher (up to four-fold change) in placenta of f*WT*(*hsEng^+^*) mice as compared with f*WT*(*WT*) after 13 and 18 days of pregnancy (Figure 3f,g).

**Figure 4:**
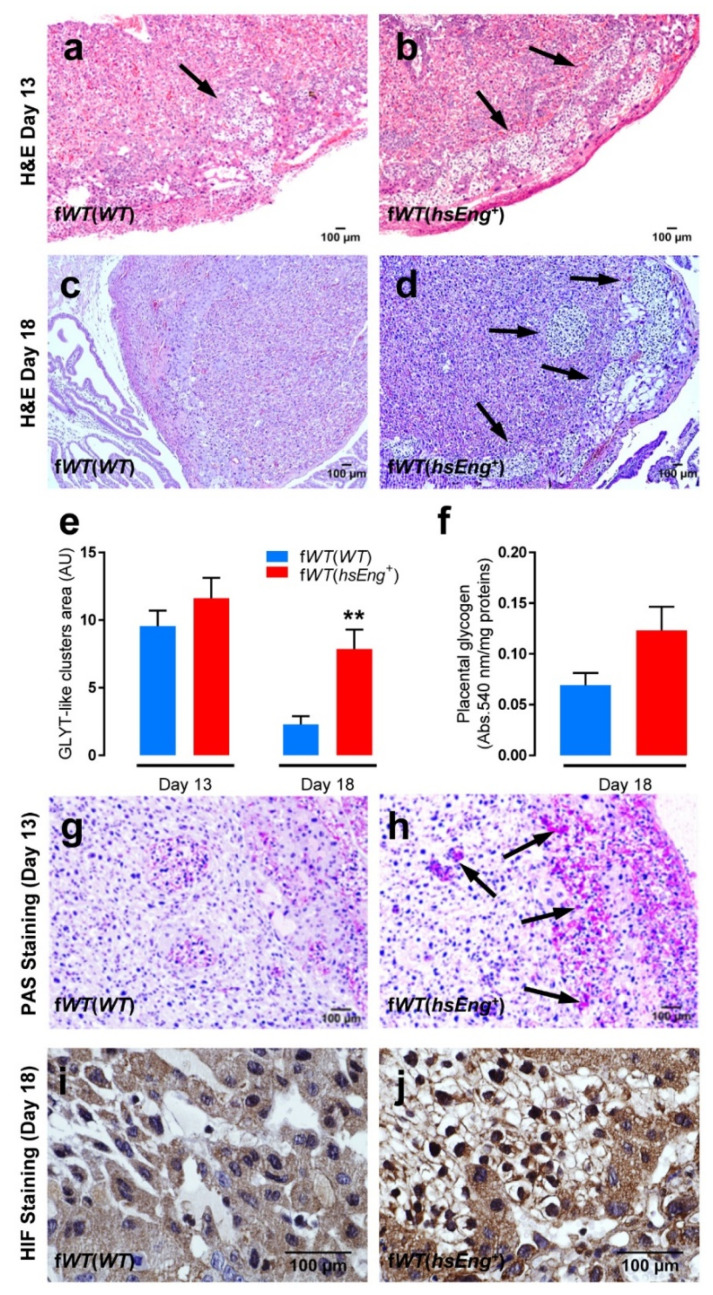
Histological alterations of placentas from f*WT*(*hsEng^+^*) mice. (**a**–**d**) Hematoxylin-eosin (H&E) staining of placentas from f*WT*(*WT*) and f*WT*(*hsEng^+^*) mice at Day 13 (**a**,**b**) and Day 18 (**c**,**d**) showing the presence of clusters of glycogen-containing trophoblast (GLYT)-like cells in the basal area of some placentas (arrows); (**e**) Quantification of the area occupied by GLYT-like clusters in placentas from f*WT*(*WT*) and f*WT*(*hsEng^+^*) mice at Day 13 (f*WT*(*WT*) *n* = 20 and f*WT*(*hsEng^+^*) *n* = 17) and Day 18 (f*WT*(*WT*) *n* = 13 and f*WT*(*hsEng^+^*) *n* = 16) of gestation; (**f**) Glycogen content in placentas from f*WT*(*WT*) (*n* = 13) and f*WT*(*hsEng^+^*) (*n* = 15) mice at Day 18 of gestation; (**g**,**h**) Periodic acid–Schiff (PAS) staining of placentas from f*WT*(*WT*) and f*WT*(*hsEng^+^*) mice at Day 18 colocalizing high levels of polysaccharides with GLYT-like clusters; (**i**,**j**) Immunohistochemical staining for HIF-1α of placentas from f*WT*(*WT*) and f*WT*(*hsEng^+^*) mice at Day 18 showing nuclear HIF-1α expression in GLYTs from f*WT*(*hsEng^+^*). Mean ± SEM are displayed. ** *p* < 0.01 of f*WT*(*hsEng^+^*) vs. f*WT*(*WT*).

We also analyzed whether sEng induces morphologic changes in placentas. At Day 13, no relevant changes were observed when comparing placentas from f*WT*(*hsEng^+^*) and f*WT*(*WT*) mice (Figure 4a,b). However, at the end of gestation (Day 18), an increased number of cell clusters characterized by big cells with a vacuolated cytosol were observed in f*WT*(*hsEng^+^*) as compared with f*WT*(*WT*) mice placentas (Figure 4d, arrows). These accumulations of clear cells were observed mainly in the basal area of the placenta in both f*WT*(*hsEng^+^*) and f*WT*(*WT*) placentas at Day 13 (Figure 4a,b, arrows), but were rarely seen in f*WT*(*WT*) mice at Day 18 of pregnancy (Figure 4c). The area occupied by these clear cells in the placenta at Day 13 of pregnancy was similar in f*WT*(*hsEng^+^*) and f*WT*(*WT*) pregnant females. Nonetheless, at Day 18, a marked decrease (with a reduction of 80%) in the area covered by clear cells was observed in f*WT*(*WT*) placentas, whereas only a slight decrease (reduction of 33%) was observed in f*WT*(*hsEng^+^*) placentas. Thus, the area occupied by clear cells at Day 18 was significantly higher in f*WT*(*hsEng^+^*) than in f*WT*(*WT*) females (Figure 4e).

Interestingly, the size and appearance of these clear cells suggest that they are glycogen-containing trophoblasts (GLYTs). GLYTs are trophoblast-type cells of unknown origin that appear in the junction zone of the murine placenta. In a normal murine pregnancy, GLYTs begin to accumulate glycogen and can be found as tightly packed clusters embedded within spongiotrophoblast cells until 12.5 days of gestation, while their number steadily decrease until the end of pregnancy [40,41,42]. However, in our f*WT*(*hsEng^+^*) model of preeclampsia, the number of these GLYT-like clusters at the end of pregnancy was markedly higher than in f*WT*(*WT*) mice. In order to confirm that these highly vacuolated cells were indeed GLYT, we performed periodic acid-Schiff (PAS) stain in placental slides. In fact, polysaccharide accumulations, most probably glycogen, were observed in the cytoplasm of these cells (Figure 4g,h). To further demonstrate that these cells were indeed GLYTs, the content of glycogen in placentas was measured at Day 18 of gestation. We observed that the amount of glycogen tended to be higher in f*WT*(*hsEng^+^*) than in f*WT*(*WT*) females’ placentas, although this difference did not reach statistical significance (Figure 4f).

There are accumulating data to suggest that changes in placental glycogen deposition is a hallmark feature of a compromised pregnancy associated with fetal growth restriction and, paradoxically, with fetal overgrowth [43]. Overall, the above data support the hypothesis that high plasma levels of sEng present in our animal model induce structural placental alterations. 

Finally, we analyzed whether high plasma levels of sEng were associated with placental hypoxia. Under hypoxic conditions, HIF-1α migrates to the nuclei, therefore, we evaluated the nuclear staining of HIF-1α by immunohistochemistry. Counterstaining with hematoxylin allowed the visualization of blue nuclei, as well as the accumulations of clear cells previously characterized as GLYT cells. Nuclear HIF-1α appearance at Day 13 was infrequent in most placental cells of both types of animals, f*WT*(*hsEng^+^*) and f*WT*(*WT*) (data not shown). However, at Day 18, the nuclear expression of HIF-1α was very prominent in certain areas of f*WT*(*hsEng^+^*) placentas, while it remained infrequent in f*WT*(*WT*). Interestingly, these hypoxic cells were restricted to the clusters of GLYT cells that are rare in f*WT*(*WT*) placentas (Figure 4i,j).

### 2.4. Soluble Endoglin Modifies Cytotrophoblast Cell Biology

**Figure 5:**
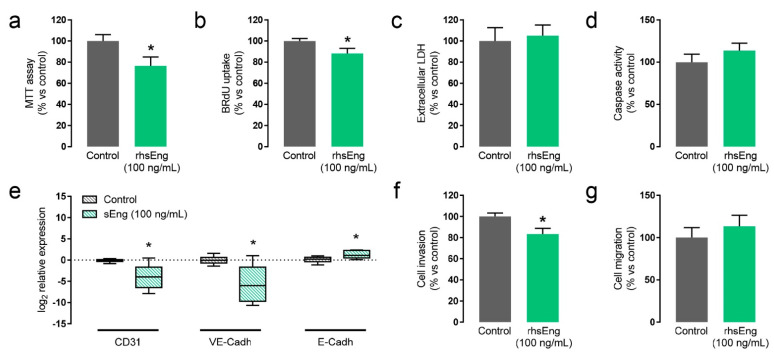
Soluble endoglin modifies cytotrophoblast cell biology. (**a**,**b**) Effect of rhsEng on JAr cell proliferation measured by MTT assay after 5 days in culture (*n* = 5) (**a**) or BrdU incorporation during 2.5 h (**b**) (*n* = 5); (**c**) Effect of rhsEng on trophoblast cell necrosis evaluated measuring extracellular levels of lactate dehydrogenase (LDH) of JAr cells treated or not with rhsEng (*n* = 4); (**d**) Caspase activity in cell lysates of JAr cells treated or not with rhsEng (*n* = 7); (**e**) qRT-PCR analysis of PECAM1, VE-cadherin, and E-cadherin expression in human placental extracts treated or not with rhsEng (*n* = 6); (**f**) Quantification of JAr invasiveness through the Matrigel^®^-coated transwell in fetal bovine serum (FBS) gradient, with or without rhsEng treatment (*n* = 6); (**g**) Quantification of JAr migration through the uncoated transwell in FBS gradient, with or without rhsEng treatment (*n* = 5). Mean ± SEM are displayed. * *p* < 0.05 of 100 ng/mL of rhsEng vs. control.

To further study the effect of sEng in placental functions, we used a human trophoblast cell line (JAr cells) and human placental explants. JAr cells were treated with 100 ng/mL of recombinant human soluble endoglin (rhsEng) and cell proliferation, migration, invasion, and death were analyzed. We observed that treatment with rhsEng significantly reduced cell proliferation in about 10–20%, as measured by MTT uptake (Figure 5a) and BrdU incorporation (Figure 5b). In order to confirm that these differences were not due to an increment of cell death, cell necrosis and apoptosis were evaluated. Cell necrosis was assessed by measuring the release of lactate dehydrogenase (LDH) to the culture medium. The amount of LDH released by either rhsEng-treated or non-treated cell was similar, suggesting that sEng does not induce trophoblast necrosis (Figure 5c). Moreover, cell apoptosis was assessed measuring the activity of the effector caspase-3 after 48 h stimulation with rhsEng. Our results showed that levels of activated caspase 3 were similar in the cells treated or not with rhsEng (Figure 5d). Therefore, the inhibitory effect of sEng on cell proliferation was not due to increased cell death.

A key function of trophoblasts is to collaborate in the remodeling of the spiral arteries, and alterations in this process are a common feature of pregnancy pathologies such as preeclampsia [40]. It has been described that trophoblasts undergo pseudovasculogenesis, a switching process by which the trophoblast changes its phenotype, reducing epithelial receptors and acquiring endothelial-related receptors [41]. This pseudovasculogenesis process is associated with trophoblast invasiveness, which is necessary for the correct remodeling of spiral arteries, and is defective in preeclampsia, further suggesting its functional importance [41,42,43]. 

Thus, we analyzed the effect of sEng on the pseudovasculogenesis of trophoblasts in placental explants. We found that treatment with 100 ng/mL of rhsEng led to decreased PECAM1 and VE-cadherin and increased E-cadherin mRNA expression, endothelial and epithelial cell receptors, respectively (Figure 5e). These results suggest that the pseudovasculogenesis process is impaired in the presence of sEng and that the resulting phenotype is similar to that of invasive cytotrophoblasts in preeclampsia [41]. Since pseudovasculogenesis is also linked with trophoblast invasion, we assessed the effect of sEng in this process using a transwell cell migration assay. Invasiveness of JAr cells through Matrigel^®^ was significantly diminished after addition of rhsEng (Figure 5f). This inhibitory effect was dependent on the presence of extracellular matrix, since a similar experiment without Matrigel^®^ showed no differences between treated or untreated cells (Figure 5g).

## 3. Discussion

The presence of high circulating levels of sEng in preeclampsia has been widely described, and therefore it has been proposed as an early diagnostic and prognostic biomarker of this disease [18,44,45]. Moreover, several studies in animal models have demonstrated that increased plasma levels of sEng could play a pathogenic role in preeclampsia [14,18,24,26]. However, the animal models used up to date might not be wholly representative of the actual disease, as the symptoms associated with increased plasma levels of sEng in those models were not dependent on gestation, and only phenocopied the so-called second step of the disease, related to the systemic mother’s response and associated clinical symptoms [46]. Thus, we have developed a murine model of preeclampsia in which plasma levels of sEng are normal before pregnancy, but they increase during the pregnancy, as in the human disease. In our experimental model, pregnant f*WT*(*hsEng^+^*) mice showed an increase in plasma hsEng levels from the 11th day of gestation and this increase was maintained until the end of pregnancy. This timing is similar to that of human preeclampsia because placentation in mice ends on Day 10.5 and it is equivalent to the beginning of the second trimester of gestation in humans [47] and it is at this time point when plasma sEng levels are increased in human preeclampsia [37,44]. 

In our mouse model of preeclampsia, pregnant f*WT*(*hsEng^+^*) mice present progressive hypertension and proteinuria, as well as fetal growth reduction, which are typical features of preeclampsia [47]. While plasma levels of sEng were already increased halfway through the gestation period, both arterial pressure and proteinuria were higher in hsEng*^+^* than in WT pregnant females only in the last week of gestation. Thus, in this experimental model of preeclampsia, plasma levels of sEng maintained their role as predictive biomarkers of the disease and they were confirmed as a contributor for blood pressure increase and proteinuria development. Moreover, our results suggest that the contribution of high levels of sEng to the preeclampsia phenotype is not immediate but needs a sustained increase of plasma sEng. Interestingly, we recently described that sEng induced the expression of BMP4 which, in turn, mediated the sEng-dependent effect in hypertension [26]. The existence of this intermediate may account for the observed delay between the appearance of increased plasma sEng levels and an increase in blood pressure. 

The ratio between plasma levels of sFlt1 and PlGF is also increased before the onset of human preeclampsia and, together with the plasma concentration of sEng, has been proposed to be a useful biomarker for diagnosis and prognosis of preeclampsia [17,36]. Similarly, in our model of f*WT*(*hsEng^+^*) mice, higher plasma levels of endogenous sEng and higher sFlt1/PlGF ratios were observed as compared with f*WT*(*WT*) pregnant mice, supporting its validity as a model of preeclampsia. The increase of plasma levels of sEng has been described in different animal models of preeclampsia such as placental ischemia in pregnant rats due to the reduction of uterine perfusion (RUPP) [7] or feto-placental expression of the transcription factor STOX1 in transgenic mice [48], both characterized by the presence of placental abnormalities [49]. Furthermore, several in vitro studies have shown an increased release of sEng by trophoblasts subjected to hypoxia, oxidative stress, or inflammation [14,50,51]. Interestingly, our results show that placentas of f*WT*(*hsEng^+^*) mice present continuous inflammation together with an early increase of oxidative stress. While we did not find a reduction in placental blood supply in our animal model, these placental alterations may, based on the above results, have entailed a further increase in sEng levels and in the sFlt1/PlGF ratio. 

The defective development of the placenta plays a key role in the origin of preeclampsia [4,52]. Thus, the disease is apparently associated with a defective remodeling of uterine spiral arteries that maintains these vessels with a low capacitance, impeding the increase of blood flow to the placenta that is necessary for a healthy pregnancy [53,54,55]. Arterial remodeling occurs when extravillous cytotrophoblasts (EVCT), which are around the spiral arteries, invade the vessel wall and reach their inner face, temporarily substituting the endothelial cells and leading to vessels with lower resistance, and thus higher blood flow, in a process called pseudovasculogenesis [41,53,54,55]. This process is associated with changes in the expression of adhesion molecules such as cell adhesion molecules (CAMs), integrins, and cadherins. Thus, EVCT, with an epithelial phenotype defined by the expression of E-cadherin and β4 and β5 integrins, becomes invasive and differentiates to endovascular cytotrophoblasts (enCT) with a more endothelial phenotype, characterized by the expression of VE-cadherin, CAMs, as well as α1 and β3 integrins [41]. On the contrary, in preeclampsia, where this process is altered, preeclamptic placentas present a strong staining of E-cadherin and a low staining of VE-cadherin and PECAM resulting in less invasive cytotrophoblasts [41,42,43]. Compatible with these findings, our results showed that human placental explants incubated with sEng have increased expression of E-cadherin, as well as lower expression of PECAM1 and VE-cadherin as compared with explants incubated without sEng. Moreover, the presence of sEng reduced both proliferation and invasiveness of the trophoblast cell line JAr. Hence, it can be speculated that high sEng levels modify the pseudovasculogenesis process, maintaining the epithelial phenotype, while weakening the endothelial-like phenotype of the trophoblasts, and thus reducing their invasive ability. In this regard, the presence of GLYTs clusters at the end of gestation in f*WT*(*hsEng^+^*) mice could be related to deficient trophoblasts migration/invasion to the maternal decidua, and thus to impaired pseudovasculogenesis, as observed in our in vivo and in vitro studies. In the rodent’s placenta, GLYT cells share a common lineage with the EVCT [56,57]. Around the seventh day of gestation, GLYTs appear in the junctional zone (JZ) of the placenta [58,59]. From the 10th to the 12th day, GLYTs begin to accumulate glycogen and can be found as clusters embedded within spongiotrophoblasts (SpT). After the 13th day, GLYTs begin to invade the interstitium migrating into the maternal decidua and congregate around spiral arteries [57,60,61,62]. In our experimental model, a decrease in the number of GLYT clusters in the placenta in the last days of gestation in pregnant f*WT*(*WT*) but not in f*WT*(*hsEng^+^*) mice was observed. These GLYT clusters present high nuclear expression of HIF-1α suggesting that they are undergoing hypoxia. However, this expression pattern of HIF-1α was not observed in the surrounding trophoblasts, suggesting that hypoxia occurs only in the GLYTs. Of note, when analyzed by laser Doppler, no statistically significant differences, in fetal or in placental blood flow which could explain an ischemic setting, were observed between f*WT*(*hsEng^+^*) and control mice. Thus, we hypothesize that a discrete small reduction in total placental blood flow in the f*WT*(*hsEng^+^*) mice may not be enough to induce hypoxia in the spongiotrophoblasts, with a low metabolic rate, but may be sufficient to induce hypoxia in the GLYTs, that are cells metabolically very active [43]. However, further experiments, which are beyond the scope of this study, would be needed to fully test this hypothesis. In preeclampsia, the onset of placental hypoxia leads to the appearance of oxidative stress, which may cause damage to proteins, lipids, or DNA, as well as endothelial dysfunction and endovascular systemic inflammation [63]. In line with the literature, we found an increase in lipid peroxidation that occurred earlier in f*WT*(*hsEng^+^*) than in f*WT*(*WT*) pregnant mice. In addition, we observed, in the placenta of f*WT*(*hsEng*^+^) mice, an increased expression of proinflammatory cytokines such as IL-6 and IL-1β as compared with f*WT*(*WT*). So far, most experimental animal studies have shown that increased inflammation and oxidative stress were associated with increased release of sEng [14,50]. Therefore, increased ROS and proinflammatory cytokines observed in our model by elevated levels of sEng could promote further increases in plasma levels of sEng which, in turn, may lead to a positive feedback loop, thus, enhancing the disease progression. 

**Figure 6:**
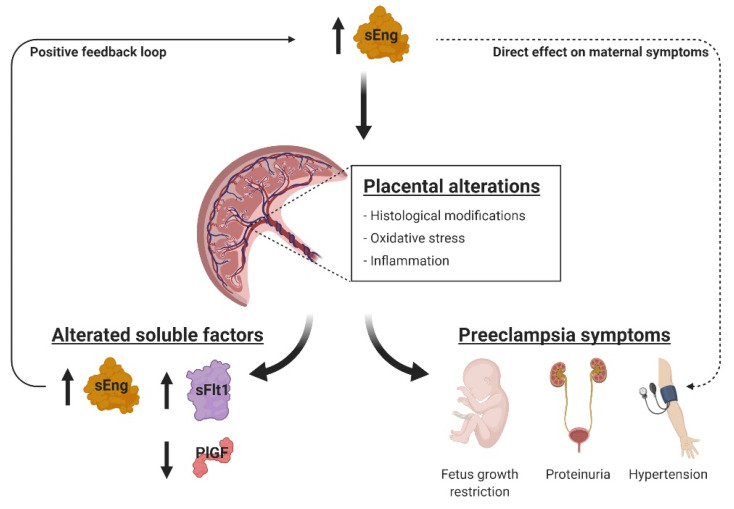
Hypothetical model of sEng role in preeclampsia. The high levels of soluble endoglin (sEng) are directly involved in the appearance of maternal preeclampsia symptoms, such as hypertension or proteinuria (dashed arrowhead on the right), as shown in non-pregnant animal models. But during pregnancy, sEng can also induce placental alterations such as inflammation, oxidative stress, and hypoxia. These alterations seem to be due to a defective pseudovasculogenesis and a diminished proliferative and invasive capacity of trophoblasts, and lead to the appearance of maternal symptoms, as well as the abnormal levels of soluble factors. As sEng is one of these factors whose expression is increased, it could trigger a positive feedback loop that may contribute to aggravate the disease (continuous arrowhead on the left). Created with BioRender.com.

In conclusion, our study describes a new model of pregnancy-associated increase of sEng levels, supporting the pathogenic contribution of sEng to preeclampsia. We report, for the first time, that sEng not only participates directly in the maternal symptoms, such as hypertension or proteinuria, but also gives rise to alterations in the placental function and structure related to the development of preeclampsia. These alterations could be responsible for a further increase in the levels of sEng, thus, giving rise to a positive feedback loop that could contribute to aggravate the disease (Figure 6). According to our results, sEng appears to have a much more relevant role in preeclampsia than initially proposed, opening new therapeutic avenues of research based on sEng. It can be postulated that, while sEng per se might not be the initial trigger of preeclampsia, it may act in concert with additional pathophysiological stimuli, thus, contributing to a full disease phenotype. Overall, we believe that the development of this novel animal model represents an important advance for future research and discovery in preeclampsia.

## 4. Materials and Methods

### 4.1. Animals and Study Design

A mouse line that overexpresses human soluble endoglin (hsEng) on a CBA ×C57BL/6J background (*hsEng^+^*) was generated at the Genetically Modified Organisms Generation Unit (University of Salamanca, Spain), as previously described [14]. A breeding colony of adult *hsEng^+^* mice has been maintained in the pathogen-free facilities for genetically modified mice at the University of Salamanca and backcrossed with CBA×C57Bl/6 mice for nine generations. Routine genotyping of DNA, isolated from mouse tail biopsies, was performed by PCR using the primers previously reported [14]. For the present study, male transgenic *hsEng^+^* mice were crossed with female *wild type* (*WT*) mice (CBA×C57BL/6J background). Pregnant WT female resulting from this cross were called f*WT*(*hsEng*^+^). Pregnant mice resulting from the cross between male *WT* with female *WT* mice (CBA×C57BL/6J background), were called f*WT*(*WT*) (Figure 1). 

For animal anesthesia, 2% isoflurane in oxygen was used. During recovery from the anesthesia, heat was provided and, when necessary, a dose of buprenorphine (0.05 mg/kg) was subcutaneously administered. Animals were sacrificed by CO_2_ inhalation or cervical dislocation, depending on the age and the experiment requirements. 

All animal procedures were conducted in strict compliance with the European Community Council Directive (2010/63/EU) and Spanish legislation (RD1201/2005 and RD53/2013). The protocols were approved by the University of Salamanca Ethical Committee (Permit Number: 006–201400038812, date 22 October 2014). Animal selection was genotype based and no randomization or blinding was performed. Animals were housed under specific pathogen-free conditions at the University of Salamanca facilities (ES-119-002001 SEARMG), in a temperature-controlled room with 12 h light/dark cycle and reared on standard chow and water provided ad libitum.

### 4.2. Enzyme-Linked Immunosorbent Assays (ELISA)

Blood samples were obtained from the jugular vein of anesthetized mice in tubes containing 1 mM EDTA. Blood samples were centrifuged at 1600× *g* for 15 min at 4 °C, and plasma was collected. Concentrations of human sEng were determined by Quantikine Human Eng/CD105 (R&D Systems, Minneapolis, MN, USA). Mouse sEng concentration was determined by Quantikine Mouse Eng/CD105 (R&D Systems). Concentration of sFLT1 was determined with the Flt1 Mouse ELISA kit (Abnova, Taipei, Taiwan). PlGF concentration was measured using a Mouse PLGF ELISA kit (Abcam, Cambridge, UK). All immunoassay kits were used following the manufacturers’ instructions.

### 4.3. Arterial Blood Pressure Measurement

Systolic blood pressure was monitored in conscious pregnant mice with an automated multichannel system by using the tail-cuff method and a photoelectric sensor (Niprem 546, Cibertec, Madrid, Spain), as previously described [64,65]. Animals were previously accustomed for several days and measures were collected at 0, 6, 10, 13, 14, and 18 days of gestation.

### 4.4. Renal Function Measurement

Renal function was analyzed, as previously described [66]. Briefly, to determine glomerular filtration rate and protein levels in urine, pregnant mice were allocated in individual metabolic cages for urine sample collection during 24 h. Animals were accustomed to being kept in metabolic cages for two days and urine was collected on the third day. Plasma and urine creatinine were quantified using a commercial kit (Quantichrom^TM^ Creatinine Assay, Biossay System, Hayward, CA, USA), following the manufacturer’s instructions. Creatinine clearance (ClCr) was calculated as follows: CrU × 24 h urine output × CrP^−1^. Proteinuria was assessed by measuring urinary protein/creatinine ratio. Protein concentration was measured using the Bradford method [67]. 

### 4.5. Gene Expression Assays

For qRT-PCR analysis, total RNA was isolated using Nucleospin RNAII (Macherey-Nagel, Düren, Germany) and single-strand cDNA was generated from 500 ng of total RNA using iScript RT Supermix 5× (Bio-Rad, Hercules, CA, USA), according to the manufacturer’s instructions. qRT-PCR was performed in triplicate. Each 20 µL reaction contained 1 µL of cDNA, 400 nM of each primer, and 1x iQ SybrGreen Supermix (Bio-Rad). Standard curves were run for each transcript to ensure exponential amplification and to rule out nonspecific amplification. The reactions were run on an iQ5 Real-time PCR detection system (Bio-Rad). Gene expression results were normalized to β-actin (*Actb*) for mouse genes and to glyceraldehyde-3-phosphate dehydrogenase (*Gapdh*) for human genes. The primers used are listed in Table 1.

### 4.6. Placental and Fetal Perfusion

At Days 13 and 18 after pregnancy, animals were anesthetized as described above. A midline abdominal incision was done to expose the uterus. We assessed microvascular placental and fetal perfusion using laser Doppler imaging (moorLDI, Moor Instruments Ltd., Devon, UK)), as previously described [68]. In brief, a helium-neon laser scans the surface of the tissue and light, and ultrasound signal, back-scattered from moving erythrocytes, is shifted in frequency by an amount proportional to their velocity, according to the Doppler principle. These Doppler shifts are collected and processed by the instrument. For each scan, the computer builds up a color-coded image representing tissue perfusion in two dimensions. This relative measure of flow is expressed in arbitrary perfusion units. The laser head was positioned 25 cm from the measurement tissue area, and each scan took ~5 s. The recorded images were analyzed using dedicated image-processing software (moorLDLS, Moor Instruments Ltd., Devon, UK).

### 4.7. Histological Studies

Placenta and kidney tissue were fixed in 4% neutral-buffered formalin and subsequently embedded in paraffin. Three µm thick sections were stained with hematoxylin and eosin (H&E) using a standard protocol. Tissue sections were processed for immunohistochemistry as follows: Endogenous peroxidase was blocked with 3% hydrogen peroxide, followed by immunohistochemical staining for HIF-1α (antibody 113642, 1∶750 dilution, Abcam) as reported [69]. Periodic acid–Schiff stain (PAS) is a standard histological stain used to detect polysaccharides such as glycogen, and glycoproteins, glycolipids, and mucins in tissues. These compounds react with the Schiff reagent to produce a characteristic positive magenta color. For this stain, we used a commercial PAS staining kit (101646, Sigma-Aldrich, St. Louis, MO, USA), following the manufacturer’s instructions. Sections were analyzed by light microscopy and images were taken with an Olympus DP70 BX51 microscope (Olympus, Tokyo, Japan).

### 4.8. Placental Biochemical Determinations

For glycogen determination, freshly collected placental samples were homogenized in PBS and centrifuged at 10,000 *g* for 10 min at 4 °C. Subsequently, the glycogen concentration in that supernatant was determined and corrected for the protein concentration. The glycogen concentration was determined using the colorimetric Glycogen Assay Kit (Cell Biolabs, Inc., San Diego, CA, USA), following the manufacturer´s instructions. Protein concentration was determined by the method described above in the Section 4.4 for proteinuria measurement.

Lipid peroxidation, a useful marker for oxidative stress, was assessed as thiobarbituric acid-reactive substances (TBARS) in placental homogenates, as previously described [70], and corrected for the protein concentration of the homogenate.

### 4.9. Cell Culture and Placental Explants

The human placental choriocarcinoma cell line JAr (Lonza, Walkersville, MD, USA) and placental explants were used for in vitro experiments. The JAr cell line expresses endoglin and has been used as a model in trophoblast studies [71]. JAr cells were cultured in RPMI-1640 medium with glucose (4.5 g/L) and penicillin-streptomycin (Gibco, Thermofisher, Pittsburgh, PA, USA) in the presence of 10% fetal bovine serum (FBS) (Gibco). 

Human fresh placental explants were collected in the delivery room (University Hospital, Salamanca, Spain), within 30 min of delivery from patients without complications. After removal of decidua and large vessels, 2 cm of tissue was cultured in RPMI-1640 medium without FBS. When necessary, JAr cells and explants were incubated with 100 ng/mL recombinant human sEng (rhsEng, 1097-EN, R&D Systems, Minneapolis, MN, USA).

### 4.10. Cell Proliferation and Apoptosis Assays

For proliferation assays, 5 × 10^3^ cells per well were seeded in 24-well plates in RPMI-1640 with 10% FBS containing or not 100–200 ng/mL rhsEng at 37 °C with 5% CO_2_ for 24 h. Proliferation was determined by incubating cell cultures with 0.5 mg/mL 3-[4.5-dimethylthiazol-2-yl]-2.5-diphenyl tetrazolium bromide (MTT) (Sigma-Aldrich, St. Louis, MO, USA) for 4 h. Then, 10% SDS in 0.01M HCl was added at a 1:1 (*v*/*v*) ratio and left overnight at 37 °C. Finally, absorbance was measured at 570 nm. The effect of soluble Eng on trophoblast proliferation was also measured using a commercial BrdU (5-bromo-2-deoxyuridine) ELISA kit (Roche, Basilea, Switzerland), following the manufacturer’s instructions. 

Apoptosis was assessed by measuring activated caspase-3 with a commercial caspase-3 assay kit (Innoprot, Derio, Spain) and cell necrosis was assessed by the release of LDH to the culture medium, as measured by the ScienCell™ LDH Citotoxicity Assay (Innoprot), following manufacturer´s instructions.

### 4.11. Transwell Migration and Invasion Assays

The transwell cell migration assay measures the capability of cells to migrate through the transwell membrane, whereas the transwell cell invasion assay measures the invasion of cells through the extracellular matrix and transwell membrane. Cells marked with the cell-permeant dye calcein-AM (Sigma-Aldrich, St. Louis, MO, USA) were diluted in serum-free culture medium and plated on top of the filter membrane in a transwell with 8 µm pore size (FluoroBlock™, Corning, Corning, NY, USA) placed in a 24-well plate. Culture medium containing 10% FBS was added to the bottom chamber and plates were incubated at 37 °C and 5% CO_2_ to allow the cells to settle down. For invasion assays, Matrigel^®^ (R&D Systems, Minneapolis, MN, USA) was loaded on top of the transwell membrane and cells were added on top of the Matrigel coating to simulate invasion through the extracellular matrix. The fluorescence was measured at the bottom of the plate at 8 h (migration assays), and at 24 h for (invasion assays), and then normalized against the control condition. 

### 4.12. Data Presentation and Statistical Analysis

Images shown are representative and all data, except those obtained by qRT-PCR, are expressed as the mean ± SEM. qRT-PCR results are represented in box plots that show the median and the 25th–75th percentiles, with whiskers showing the 10th–90th percentiles. In vitro experiments were repeated at least three times. For data related to controls, such as qRT-PCR, log transformation was performed before the statistical study for normalization. The D’Agostino–Pearson normality test was applied to the datasets prior to statistical comparisons. The Kolmogorov–Smirnov test was used for small datasets. For normally distributed datasets, Student’s t-test was used. The Mann–Whitney U test was used as the nonparametric test. One-way or two-way ANOVA was used to assess the differences between groups in the time-course experiments. Sidak’s post-hoc test was used after ANOVA. The following three levels of significance were defined: * *p* < 0.05, ** *p* < 0.01, and *** *p* < 0.001. All analyses and graphs were performed using GraphPad Prism version 7.0.0 for Windows (GraphPad Software, San Diego, California USA, www.graphpad.com).

## Figures and Tables

**Table 1:** Primer sequences.

Gene (Protein)	qPCR Primers	Organism
*ENG* (endoglin)	Forward-AGGTGCTTCTGGTCCTCAGT Reverse-CCACTCAAGGATCTGGGTCT	Human
*Il1b* (Interleukin-1β)	Forward-GCCTGTGTTTTCCTCCTTGC Reverse-TGCTGCCTAATGTCCCCTTG	Mouse
*Il6* (Interleukin-6)	Forward-TCCAGTTGCCTTCTTGGGAC Reverse-AGTCTCCTCTCCGGACTTGT	Mouse
*Actb* (β-actin)	Forward-TCTACAAATGTGGCTGAGGACT Reverse-GAGGGACTTCCTGTAACCACTT	Mouse
*CADH1* (E-cadherin)	Forward-CAAGCTATCCTTGCACCTCAG Reverse-GCATCAGAGAACTCCTATCTT	Human
*CADH5* (VE-cadherin)	Forward-TGGTCACCCATGCATCTTCC Reverse-CCATGACGAAGGGTGAGCTT	Human
*PECAM1* (CD31)	Forward-TGCCGTGGAAAGCAGATAC Reverse-GGAGCAGGGCAGGTTCATAA	Human
*GAPDH*	Forward-CAATGACCCCTTCATTGACC Reverse-GACAAGCTTCCCGTTCTCAG	Human

## Data Availability

The data presented in this study are available on request from the corresponding author.

## Supplementary Materials

The following are available online at https://www.mdpi.com/1422-0067/22/1/165/s1, Figure S1: Renal function in pregnant f*WT*(*hsEng^+^*) mice, Figure S2: Pregnant f*WT*(*hsEng^+^*) mice present reduced fetal weight and number of viable fetuses.

## Abbreviations

BMP4	Bone morphogenetic protein 4
CAMs	Cell adhesion molecules
BrdU	5-Bromo-2-deoxyuridine
enCT	Endovascular cytotrophoblasts
Eng	Endoglin
EVCT	Extravillous cytotrophoblasts
FBS	Fetal bovine serum
f*WT*(*h**sEng*^+^)	Pregnant *WT* females bearing both *WT* and *h**sEng^+^* fetuses
f*WT*(*WT*)	Pregnant *WT* females bearing only *WT* fetuses
GLYT	Glycogen-containing trophoblast
H&E	Hematoxylin and eosin
HIF-1α	Hypoxia-inducible factor 1 alpha
hsEng	Human soluble endoglin
IL	Interleukin
LDH	Lactate dehydrogenase
MMP	Matrix metalloprotease
msEng	Murine soluble endoglin
MTT	3-[4.5-Dimethylthiazol-2-yl]-2.5-diphenyl tetrazolium bromide
PAS	Periodic acid-Schiff
PlGF	Placental growth factor
qRT-PCR	Quantitative reverse transcription polymerase chain reaction
rhsEng	Recombinant human soluble endoglin
ROS	Radical oxygen species
RUPP	Reduction of uterine perfusion
sEng	Soluble endoglin
SEM	Standard error of the mean
sFlt1	Fms-like kinase 1
SpT	Spongiotrophoblasts
VEGF	Vascular endothelial growth factor
WT	Wild type
