# Supplementary material for: Pregnancy-Induced High Plasma Levels of Soluble Endoglin in Mice Lead to Preeclampsia Symptoms and Placental Abnormalities
Source: Int J Mol Sci. 2020 Dec 26;22(1):165. doi: 10.3390/ijms22010165 (PMC7795873; doi:10.3390/ijms22010165)
Supplement: Supplementary file 1 [file ijms-22-00165-s001.pdf]

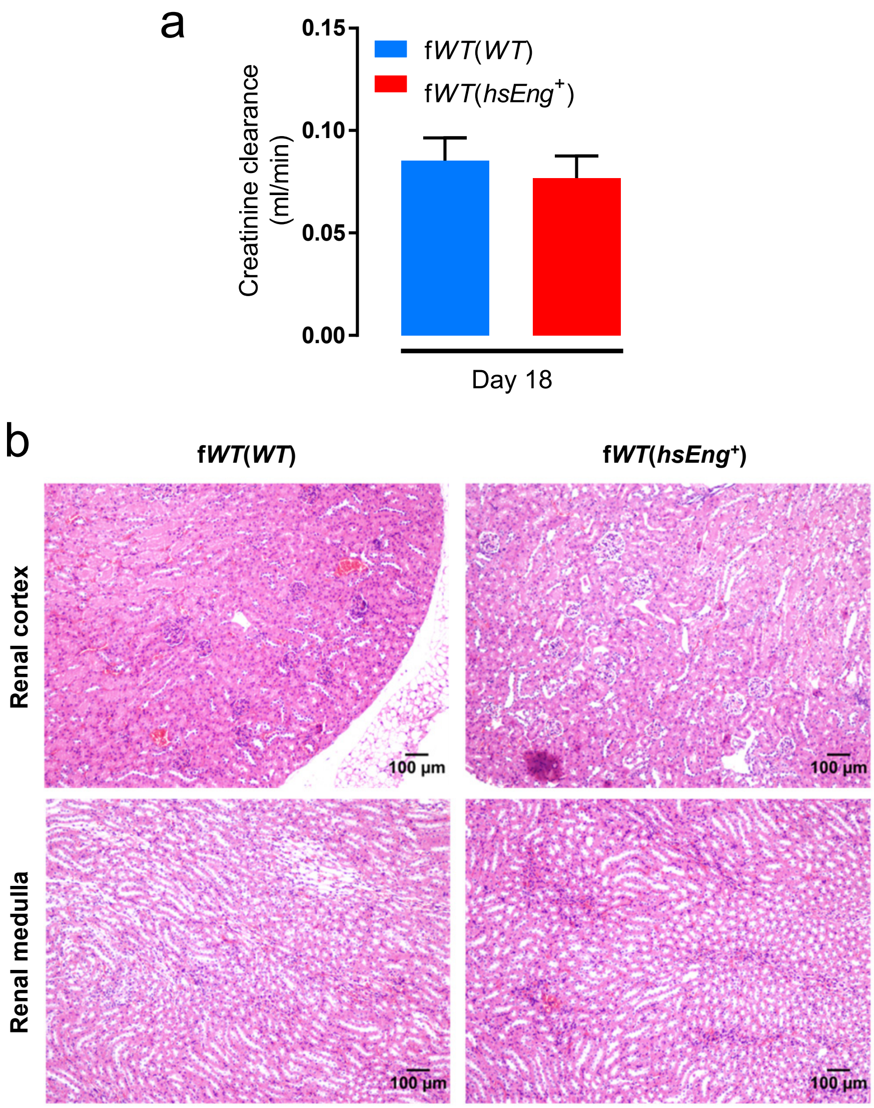

Figure S1. Renal function in pregnant f*WT*(*hsEng*⁺) mice. (a) Creatinine clearance of f*WT*(*WT*) and f*WT*(*hsEng*⁺) at day 18 of gestation [fWT(WT) n=7 and fWT(hsEng+) n=10]. (b) Hematoxylin-eosin staining of kidneys from f*WT*(*WT*) and f*WT*(*hsEng*⁺) mice at day 18. No significant alterations are observed in the histological study in either the renal cortex or the renal medulla. Mean ± SEM are displayed.

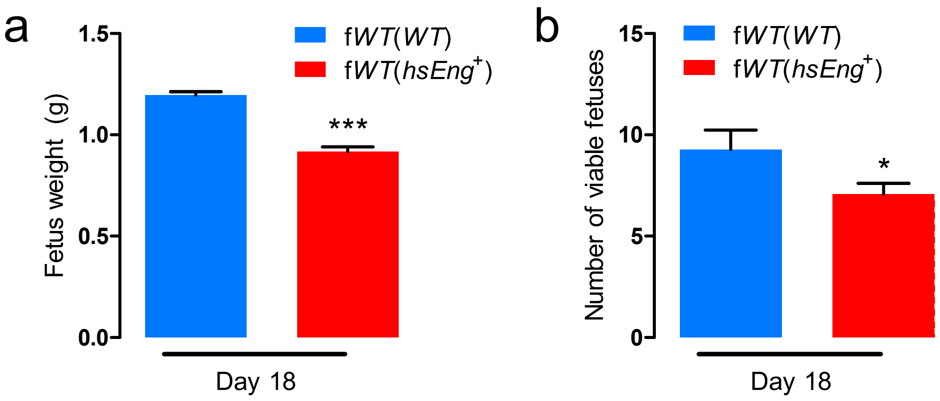

Figure S2. Pregnant f*WT*(*hsEng*$^+$) mice present reduced fetal weight and number of viable fetuses. (a) Fetus weight of f*WT*(*WT*) and f*WT*(*hsEng*$^+$) at day 18 of gestation [fWT(WT) n=103 and fWT(hsEng+) n=99]. (b) Number of viable fetuses per litter from f*WT*(*WT*) and f*WT*(*hsEng*$^+$) [fWT(WT) n=11 and fWT(hsEng+) n=14]. Mean ± SEM are displayed. *p<0.05; ***p<0.001 of f*WT*(*hsEng*$^+$) vs f*WT*(*WT*).
